# Quality of Life after Risk-Reducing Hysterectomy for Endometrial Cancer Prevention: A Systematic Review

**DOI:** 10.3390/cancers14235832

**Published:** 2022-11-26

**Authors:** Samuel Oxley, Ran Xiong, Xia Wei, Ashwin Kalra, Michail Sideris, Rosa Legood, Ranjit Manchanda

**Affiliations:** 1 Wolfson Institute of Population Health, Queen Mary University of London, Charterhouse Square, London EC1M 6BQ, UK; 2 Department of Gynaecological Oncology, Barts Health NHS Trust, London EC1A 7BE, UK; 3 Department of Women’s Health, Queen Elizabeth Hospital, Lewisham and Greenwich NHS Trust, London SE18 4QH, UK; 4 Department of Health Services Research and Policy, London School of Hygiene & Tropical Medicine, London WC1H 9SH, UK; 5 MRC Clinical Trials Unit at UCL, Institute of Clinical Trials & Methodology, Faculty of Population Health Sciences, University College London, London WC1V 6LJ, UK; 6 Department of Gynaecology, All India Institute of Medical Sciences, New Delhi 110029, India

**Keywords:** endometrial cancer prevention, risk reducing hysterectomy, hysterectomy, quality of life, satisfaction, decision regret, menopause, Lynch syndrome

## Abstract

**Simple Summary:**

This study aimed to review the research on the quality of life after hysterectomy (surgical removal of the womb) to prevent endometrial (womb) cancer. We conducted a systematic review on this topic, and found only four papers. After this operation, women had reduced worry about cancer, and were generally satisfied. There was a severe impact from the removal of both ovaries, which resulted in menopause-symptoms, particularly for women not taking hormone-replacement. We also looked at papers reporting quality of life after hysterectomy to treat heavy or abnormal periods. We found 25 papers, and generally these reported that women were satisfied after this operation, with improved quality of life, and were very unlikely to have worse sexual function or urinary problems. This review demonstrates that there is very little evidence on quality of life after this surgery to prevent endometrial cancer, and highlights how more research is needed.

**Abstract:**

Background: Risk-reducing hysterectomy (RRH) is the gold-standard prevention for endometrial cancer (EC). Knowledge of the impact on quality-of-life (QoL) is crucial for decision-making. This systematic review aims to summarise the evidence. Methods: We searched major databases until July 2022 (CRD42022347631). Given the paucity of data on RRH, we also included hysterectomy as treatment for benign disease. We used validated quality-assessment tools, and performed qualitative synthesis of QoL outcomes. Results: Four studies (64 patients) reported on RRH, 25 studies (1268 patients) on hysterectomy as treatment for uterine bleeding. There was moderate risk-of-bias in many studies. Following RRH, three qualitative studies found substantially lowered cancer-worry, with no decision-regret. Oophorectomy (for ovarian cancer prevention) severely impaired menopause-specific QoL and sexual-function, particularly without hormone-replacement. Quantitative studies supported these results, finding low distress and generally high satisfaction. Hysterectomy as treatment of bleeding improved QoL, resulted in high satisfaction, and no change or improvements in sexual and urinary function, although small numbers reported worsening. Conclusions: There is very limited evidence on QoL after RRH. Whilst there are benefits, most adverse consequences arise from oophorectomy. Benign hysterectomy allows for some limited comparison; however, more research is needed for outcomes in the population of women at increased EC-risk.

## 1. Introduction

Endometrial cancer (EC) affects over 417,000 people per year [1], and is predicted to increase by 50% by 2040 [2]. Whilst the average lifetime risk is around 3%, some individuals are at increased risk due to genetic factors, which include Lynch Syndrome (up to 57% [3]), Cowden syndrome (up to 28%) [4], an affected family history [5], and numerous single nucleotide polymorphisms (SNPs) [6]. Additionally, non-genetic risk factors include obesity, type-2 diabetes, nulliparity and Tamoxifen use [7,8,9]. Prediction models are being developed to provide individuals with personalised risk scores incorporating these factors [10,11].

For women at substantially increased EC risk, such as those with Lynch syndrome, EC prevention is highly cost-effective for health systems [12,13] and recommended by international guidelines [14]. The most effective prevention is surgical removal of the uterus in the form of risk-reducing hysterectomy (RRH), which prevents 100% of EC cases [15]. RRH is commonly performed in combination with removal of the fallopian tubes and ovaries (bilateral salpingo-oophorectomy—BSO), as most women with Lynch syndrome who undergo this surgery are also at increased risk of ovarian cancer (OC) [16].

Hysterectomy can be performed abdominally in the form of total abdominal hysterectomy (TAH), vaginally, laparoscopically either as laparoscopically assisted vaginal hysterectomy (LAVH) or total laparoscopic hysterectomy (TLH), or total robotic hysterectomy (TRH) [17,18]. In the modern era, a minimally invasive technique (TLH or TRH) is the gold-standard in suitable patients [14,19], although variation exists due to surgeon preference and training, and between different regions [20].

RRH may cause a number of potential adverse consequences, including the inability of pre-menopausal women to carry further pregnancies, and a possible detrimental impact on quality of life (QoL) [21,22]. RRH can affect sexual and bladder function due to anatomical proximity of the uterus in relationship to the vagina and the bladder. Pre-menopausal BSO results in surgical menopause which refers to a premature loss of ovarian function, leading to associated vasomotor and cognitive symptoms, and a negative impact on sexual function [23]. Surgical menopause is also associated with long-term increased risk of cardiovascular disease and osteoporosis [24]. These symptoms and long-term risks can be mitigated through the use of hormone-replacement therapy (HRT), which is recommended in such cases until the age of natural menopause provided no contraindications exist [25].

QoL is a multi-dimensional construct which accounts for objective and subjective measures of wellbeing [26]. Understanding the impact of any cancer prevention intervention on QoL is critical for patients and clinicians in deciding whether to undertake a procedure, as the QoL impact must be weighed against the benefits of reduction in cancer risk and worry. Validated assessment tools such as EQ-5D [27] allow objective quantification of QoL using utility scores, on a scale 0–1 with 1 representing perfect health and 0 death. This further allows for the calculation of quality adjusted life years (QALYs), and health-economic modelling of preventive strategies [28]. Previous health economic modelling studies on EC prevention have lacked high-quality data on utility scores of RRH, using estimates rather than values derived directly from patients who have undergone RRH, and results were sensitive to these (estimated) utility scores [13,29]. Therefore, determining the QoL after RRH remains a priority. This will advance research focused on further determining optimal strategies for EC prevention, as well as generating evidence to effectively counsel patients. To the best of our knowledge, no previous study has attempted to systematically review or synthesise evidence of QoL after RRH.

The primary aim of this systematic review is to summarise published evidence on the QoL impact of RRH for EC prevention. Secondary aims are to compare QoL outcomes in RRH-alone vs. RRH and BSO, across different types of surgical routes. Finally, in order to obtain more robust estimates on QoL post hysterectomy, we aimed to include and summarise studies that report on the impact of hysterectomy on QoL (with or without BSO) when performed for selected benign gynaecological diseases (any surgical route).

## 2. Materials and Methods

We followed a prospectively registered protocol (PROSPERO CRD42022347631) and reported in line with Preferred Reporting Items for Systematic Reviews and Meta-analyses (PRISMA) [30].

### 2.1. Literature Search

We searched PubMed, Medline & Embase from inception to July 2022 using a pre-defined search strategy (File S1). This strategy was validated [30] by evaluating whether it could identify a set of four clearly eligible studies identified on preliminary searches [31,32,33,34]. Additionally, reference lists from relevant primary studies and review articles were manually searched (including systematic reviews of hysterectomy for other indications), and researchers with expertise in the area consulted.

### 2.2. Study Selection (Inclusion Criteria)

**Figure 1:**
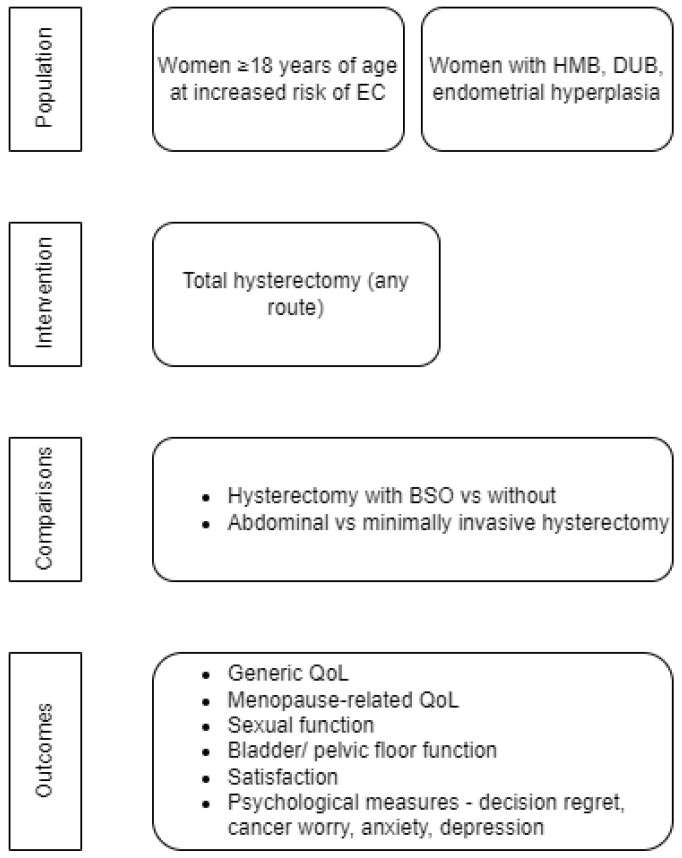
PICO framework of the systematic review. EC, Endometrial cancer; HMB, Heavy menstrual bleeding; DUB, Dysfunctional uterine bleeding; BSO, Bilateral salpingo-oophorectomy; QoL, Quality of Life.

We followed a Population Intervention Comparator Outcomes (PICO) framework to specify our inclusion criteria (Figure 1). Our primary population was defined as women ≥18 years of age at increased risk of EC (including Lynch Syndrome).

### 2.3. Using a Surrogate Population Model with Alternative Surgical Indication

As we anticipated, and confirmed by our preliminary searches, there is slim evidence on QoL after RRH for EC prevention. Hence, we prospectively sought an alternative population model to investigate our outcomes, with as near homogeneity as possible to that undergoing risk-reduction. On that basis, we included studies describing women undergoing hysterectomy as a treatment for selected gynaecological conditions, where the procedure may be similar to RRH. We considered hysterectomy for the following indications as suitable: women with heavy menstrual bleeding (HMB including fibroid related HMB)/dysfunctional uterine bleeding (DUB), and endometrial hyperplasia, as the operative procedure may be similar (including hysterectomy with and without BSO) [18,35]. Studies describing women undergoing hysterectomy predominantly for fibroids (causing pressure symptoms), endometriosis/adenomyosis and pelvic pain were excluded, as the hysterectomy procedure may be more complex than RRH, with large uteri or extra-uterine pelvic disease/adhesions, and there may be more complex and varied baseline symptomatology and hence reduced pre-operative QoL compared to women undergoing RRH for EC prevention [18,35]. This would make comparisons of post operative QoL following RRH versus this alternative indication more difficult.

However, many studies on HMB/DUB recruit women with co-existent pain disorders/pathology. On that basis, we included studies recruiting women who underwent hysterectomy for HMB/DUB if they contained a proportion of patients with pain disorders in keeping with reference (prevalence) values from the general population. We found no evidence to suggest that women with Lynch Syndrome/women undergoing RRH have a different underlying prevalence of benign gynaecological disease from the general population, and so it is reasonable to assume that a proportion of risk-reducing hysterectomies will contain co-existent endometriosis/adenomyosis. We estimated the reference prevalence of dysmenorrhoea in the general population from a systematic review [36] as 15% for severe pain [37,38], 23–37% for moderate-severe pain [39,40], and up to 76% for any pain [39]. The pooled prevalence of adenomyosis was estimated at 22.6% (95% CI 12.7–37.1%) from another systematic review [41]. The prevalence of endometriosis was estimated as 11.4% (95% CI 11.1–11.7%) when including suspected and confirmed cases by a large longitudinal study [42]. If the proportions were not specified but consecutive patients (from general population) were included, we assumed these were within the proportions of normal epidemiology.

### 2.4. Intervention

The intervention was total hysterectomy via any route (abdominal/laparoscopic/vaginal/robotic, or any combination).

### 2.5. Comparators

We aimed to compare women undergoing hysterectomy with BSO to those without BSO, separately for each population undergoing hysterectomy. We aimed to compare women having abdominal hysterectomy to those having minimally invasive surgery (including laparoscopic/robotic/laparoscopically assisted vaginal/vaginal), separately for each population.

### 2.6. Outcomes

We report studies including women undergoing RRH for EC prevention separately from studies with women undergoing hysterectomy as treatment for HMB/DUB, and endometrial hyperplasia. We included studies which reported QoL outcomes, with at least 4 months’ post-operative follow-up. This was further divided into generic QoL, menopause symptoms, sexual function, satisfaction, bladder function, and psychological measures including decision regret, cancer worry, anxiety or depression.

### 2.7. Exclusion Criteria

We excluded studies that included: (1) women who were receiving hysterectomy as part of treatment for any cancer, as cancer treatment is often more extensive than RRH, with some patients requiring more radical surgery including pelvic lymphadenectomy, and adjuvant chemo/radiotherapy. Furthermore, endometrial cancer survivors have lower QoL compared to population norms many years after treatment [43]. (2) women having treatment for any urogynaecological disease (including pelvic floor prolapse), as the reference QoL of these women may not be representative of the baseline QoL of women undergoing RRH for EC prevention, (3) similarly women having treatment solely for pelvic pain/endometriosis/adenomyosis as the primary symptom/disease, as the QoL of these women are not representative of women undergoing RRH for EC prevention (although see caveats described above), (4) women having subtotal hysterectomy (without removal of the cervix), as this treatment is not performed for risk-reduction, (5) case reports, (6) review articles (7) studies describing post-operative follow-up of under 4 months’ duration.

### 2.8. Screening of the Literature

Retrieved titles were initially transferred into reference-management software (EndNote 20.2, Clarivate-Analytics) and duplicates were removed. Two independent reviewers (SO and RX) initially screened the titles and abstracts. Following this, we retrieved the full texts of the shortlisted abstracts to assess eligibility for inclusion. Any disagreement was resolved by a third reviewer (MS) or the senior author of the study (RM). We included any study design with no restriction that follows our PICO framework and reports at least one QoL outcome. This refers to prospective or retrospective cohort studies, randomised trials or case series.

### 2.9. Data Extraction

Data were extracted in duplicate by two independent reviewers (SO and RX) using predesigned Microsoft Excel spreadsheet tables for relevant outcomes. We extracted data on study setting, design, included population, surgical intervention, reported QoL outcomes, and relevant findings for patients undergoing hysterectomy. Any differences were resolved by a third reviewer (MS) or senior author (RM).

### 2.10. Quality Assessment

Four independent reviewers (SO, RX, XW and AK) assessed the internal validity (bias specific to the study) of the included studies against validated tools. For qualitative studies we used the National Institute for Health and Care Excellence (NICE) quality appraisal checklist [44], for randomised controlled trials the Jadad score [45], and for cohort studies the Newcastle Ottawa Scale (NOS) [46].

### 2.11. Analysis

As we anticipated significant heterogeneity in the form of reporting QoL across studies, we did not intend to perform meta-analysis. Instead, we performed a qualitative synthesis of the results in structured tables which describe elements defined by our PICO framework. This includes study design, country of origin, follow-up pattern, route of hysterectomy, outcome (and relevant tool used) measured. We aimed to report extracted data in a unified (coded) way to facilitate interpretation. Coding of data was facilitated by agreement between the 3 reviewers (SO/RX/MS) and the senior author (RM).

### 2.12. Data accuracy and Availability

SO, RM, MS are the guarantors of the data quality. The corresponding and senior author (RM) made the final decision to submit for publication. All data related to this study are presented within the article and supplementary material.

### 2.13. Ethical Approval

As this is a review of existing literature, no ethical approval was required.

## 3. Results

### 3.1. Characteristics of Included Studies

**Figure 2:**
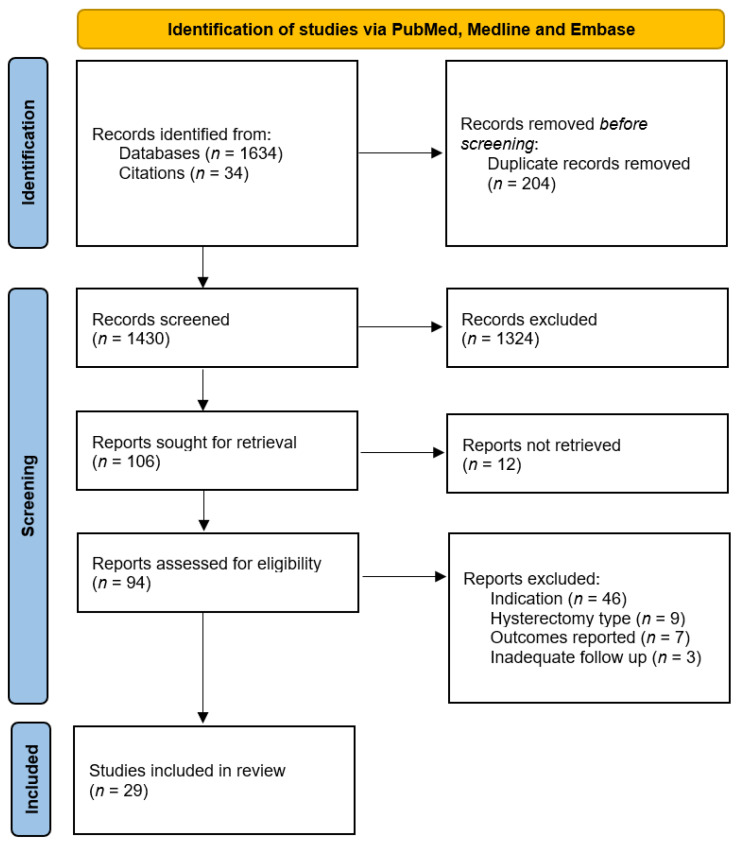
PRISMA flow diagram of study selection [30].

Figure 2 summarises the study identification and selection process.

Our initial search yielded 1634 initial citations; 19 systematic reviews [47,48,49,50,51,52,53,54,55,56,57,58,59,60,61,62] were manually checked for references yielding an additional 34 citations. Twenty-nine studies (1332 patients) were included in our qualitative synthesis—see Table 1 (characteristics of included studies). Four studies (including 64 patients) were cohort studies which investigated QoL outcomes in women undergoing RRH for Lynch Syndrome [31,32,33,34]. The mean duration of follow-up ranged from 49 to 57 months. A further 25 studies (including 1268 patients) met inclusion criteria for our additional population analogue, and described hysterectomy as a treatment of benign disease, specifically treatment of HMB/DUB [63,64,65,66,67,68,69,70,71,72,73,74,75,76,77,78,79,80,81,82,83,84,85,86,87]. Fifteen studies reported on 9 randomised controlled trials (RCTs), and 8 studies reported on separate cohort studies. Mean follow-up ranged from 6 months to 10 years.

### 3.2. Outcomes Reported

Three out of the four studies describing RRH reported analysis of qualitative interviews and two provided quantitative data (one reported both). One study reported quantitative results from validated questionnaires including General Health Questionnaire (GHQ), Menopause-specific quality of life (MENQOL), Cancer Worry Scale (CWS), and Impact of Events Scale (IES), while another study used non-validated questionnaires.

Of the 15 studies reporting hysterectomy as treatment of HMB/DUB, reported outcomes included generic QoL in 11 studies using the EQ-5D in 4 studies, EuroQol Visual Analogue Scale (VAS) in 2 studies, and the 36 item Short form survey (SF-36) in 11 studies. One study measured menopause-specific QoL using the Kupperman index (KI). Six studies measured sexual function using the McCoy sex scale (MSS) in four studies, Sexual Problems Index (SPI) in one study, Sabbatsberg Sexual Rating Scale (SSRS) in one study, Female Sexual Function Index (FSFI) in one study. Twelve studies reported on psychological outcomes including anxiety and depression with the Spielberger state trait anxiety inventory (STAI) in three studies, Beck’s Depression Inventory (BDI) in three studies and the Hospital Anxiety and Depression Scale (HADS) in three studies. Other validated and non-validated questionnaires were also used—see File S1, Table S1 for details on all outcomes measured.

### 3.3. Quality Assessment

File S1, Tables S2–S4 report in full detail on the quality assessment of the included studies. Using the NICE quality appraisal checklist, risk of bias assessment revealed low risk of bias for the 3/3 qualitative studies discussing RRH [31,32,33] (Tables S2–S4). Using the NOS, 2/2 cohort studies into RRH [31,34] revealed moderate risk of bias, due to the small sample in the assessed cohort versus the baseline population, and lack of baseline assessments. Eight cohort studies [69,72,73,74,82,83,84,87] into treatment of HMB/DUB were assessed with NOS; 7/8 studies revealed moderate risk of bias, in the lack of baseline assessments and assessments of outcome (Table S4). The Jadad scale for randomised controlled trials demonstrated moderate risk of bias in 6/9 trials [63,68,70,75,77,80,81,85,86], predominantly due to lack of clarity on blinding (Table S3). Whilst it is generally not appropriate to blind in surgical trials, very few studies commented on this, or on blinding at outcome assessment.

### 3.4. QoL following RRH

Four studies (including 64 patients) focused on QoL after RRH for EC prevention, all in the context of Lynch Syndrome [31,32,33,34]. Two papers reported pilot qualitative interviews of the same eight patients based in Canada [32,33]. The menopausal status of these patients is not provided; however, two patients were over the age of 50 at the time of surgery, and six were under 50 years. The first of these [32] focused on the decision-making experience and found that women who opted for RRH expressed no decision regret, and RRH alleviated their cancer worry. Some patients reported unexpected menopausal symptoms and felt they were insufficiently counselled pre-operatively how to mitigate these. The second paper [33] reported post-operative QoL in more detail, including “distressing and persistent” menopausal symptoms for women such as hot flushes, night sweats, skin changes, and difficulty sleeping. In addition, vaginal dryness and reduced libido resulted in a negative impact on sexual function. Those women who received adequate pre-operative counselling on HRT were, however, very satisfied with their QoL.

One UK study combined qualitative interviews with 11 patients and validated questionnaires (MENQOL, CWS, IES, GHQ) for 14 patients only [31]. Twelve patients were pre-menopausal, two patients were peri-menopausal. Qualitative analysis revealed that RRH was associated with significant reduction in cancer worry with no decision regret. Many patients took HRT and found menopausal symptoms manageable. Questionnaires revealed low rates of increased cancer worry (2/14, 14%), distress (1/14, 7%), psychiatric morbidity (1/14, 7%) or poor menopause-specific QoL (3/14, 21%). Scores for GHQ were 23.58 ± 14.40 (mean ± standard deviation), for MENQOL 9.35 ± 34.11, and for IES 8.25 ± 10.77, although no comparison is given to the age-matched population. Increased cancer worry was correlated with poor general health (r = 0.64, *p* < 0.05) and menopausal symptoms (r = 0.53, *p* < 05). Having a higher number of Lynch Syndrome-affected first-degree relatives was associated with greater psychological distress and somatic symptoms (r = 0.59, *p* < 0.05).

A Finnish study recruited 42 patients who had undergone RRH from a national Lynch Syndrome registry, and used non-validated questionnaires [34]. The median age at surgery was 42.0 years (range 32.0–67.0); the menopausal status of patients at the time of surgery is not provided. The majority (73%) of patients were generally satisfied with surgery, although 45% still described a strong fear of cancer. Further details on aspects of post-operative QoL were not reported. See Table 2 for summary of main findings.

### 3.5. QoL following Hysterectomy as Treatment for Benign Disease

Twenty-five studies including 1268 patients met inclusion criteria for our additional population analogue, and described hysterectomy as a treatment of HMB/DUB [63,64,65,66,67,72,73,74,75,76,77,78,79,80,81,82,83,84,85,86,87].

#### 3.5.1. Hysterectomy Versus Hormonal Treatment

Five studies reported outcomes of a Finnish multi-centre RCT comparing hysterectomy versus the levonorgestrel-releasing intrauterine system (LNG-IUS) for heavy menstrual bleeding [63,64,65,66,67]. In total, 117 patients were assigned to hysterectomy (TAH/vaginal/TLH), with 112 patients available for 12 month follow-up [63]. Five out of 112 patients underwent concomitant BSO. Baseline QoL (measured by EQ-5D and SF-36) was lower compared to the age-matched Finnish population, with an EQ-5D utility score of 0.78 (95% CI 0.70–0.80). Following hysterectomy, all values significantly improved by 6 and 12 months, in the case of EQ-5D by a mean increase of 0.10 (95% CI 0.06 to 0.14) at 12 months, to above the population reference. SF-36 values significantly improved, without differences between groups except for significantly lower pain scores following hysterectomy (62 (95% CI 57.6 to 66.4) at baseline, improving by 21.1 (95% CI 16.0 to 26.3) at 12 months). Menopausal symptoms at baseline and 12 months were assessed using the KI [65]. Total KI scores following hysterectomy reduced by 3.04 ± 7.96 after 12 months which was not significant (*p* > 0.05), although both hysterectomy and the LNG-IUS significantly improved insomnia, palpitations, melancholia, weakness, vertigo, myalgia and nervousness. Hysterectomy resulted in an increase in hot flushes. The effect on sexual functioning at 5-year follow-up is reported in 117 patients using the MSS and customised questionnaires [66]. Six months following hysterectomy, sexual satisfaction improved from a mean of 23.6 to 24.5, and patients reported fewer sexual problems (from a mean 4.4 to 3.8), and improved satisfaction with partner, which was maintained over the 5-year follow-up. In contrast, women receiving the LNG-IUS experienced no change to satisfaction but a decrease in partner satisfaction over this time. In total, 109 women from the hysterectomy group completed questionnaires at 10-year follow-up, by which time many had reached the menopause [67]. These results showed that sexual satisfaction (including with partner) decreased from 5 to 10 years (from a mean of 24.0 to 23.0), and sexual problems increased by a mean of 0.5, to reach 5.2, slightly greater than baseline (mean 4.5). No significant differences were seen on any measures of sexuality compared to LNG-IUS after 10 years (*p* = 0.86, 0.25, 0.30). EQ-5D scores improved after 1 to 5 years to reach a mean of 0.85 but decreased by 0.08 to baseline levels after 10 years, with no differences between groups, although the VAS decreased to a mean of 7.4 lower than baseline. Some dimensions of the SF-36 decreased from 5 to 10 years such as general health (by a mean of 8.7), although no differences were seen between groups (*p* = 0.27). Compared to baseline, no changes were seen at 10-year follow-up with measures of depression (BDI) (*p* = 0.40), or anxiety (STAI) (*p* = 0.65).

The USA multi-centre Medicine or Surgery RCT compared hysterectomy (various routes) to medical treatment (oral oestrogen and progesterone with prostaglandin synthetase inhibitor) for DUB [68]. Approximately half of this population were black ethnicity with a 63% prevalence of fibroids, and BSO was “discouraged” but rates not reported. As with the previous trial, baseline SF-36 summary scores were moderately reduced compared to population norms (Mental Component Summary (MCS) mean 45 ± 11 (SD), Physical Component Summary (PCS) mean 43 ± 8). Hysterectomy significantly increased SF-36 MCS (by a mean of 8), PCS (by a mean of 6), satisfaction with symptoms (by a mean of 44), symptom resolution (by a mean 75), sexual desire (by a mean 21), and health distress (by mean 33) at 6 months, significantly more so than medical therapy, and these improvements continued at 2-year follow-up.

A Turkish cohort study compared 32 patients treated for HMB with TAH, 37 patients with TLH, and 35 with LNG-IUS, measuring QoL using the SF-36 at 6 months’ follow-up [69]. BSO rates were not reported. There were baseline differences between groups including QoL and older age in surgical arms (mean 48.4 years in the TAH group vs. 41.3 years in the LNG-IUS group). All interventions resulted in a significant improvement in nearly all measures of QoL, with TAH improving general health from 41.6 ± 15.9 to 55.2 ± 16.0 (means ± SD) (*p* < 0.001), and TLH improving general health from 46.4 ± 16.9 to 58.3 ± 15.8 (*p* < 0.001).

#### 3.5.2. Comparison of Total vs. Subtotal Hysterectomy

A US-based multi-centre RCT compared total versus subtotal abdominal hysterectomy for the treatment of abnormal uterine bleeding including leiomyomas [70]. Sixty-four patients who underwent TAH recorded a significant improvement in all symptoms from baseline to 24-month follow-up on non-validated questionnaires including pressure/pain (from 70% to 11%), urinary urgency (from 33% to 9.4%), urge incontinence (from 18% to 3.1%), and stress incontinence (from 22% to 4.7%). Overall QoL as measured by SF-36 improved from baseline (MCS 43 ± 11, PCS 37 ± 10), to 6 months (MCS 49 ± 11, PCS 46 ± 11), with a further slight increase at 24 months (MCS 51 ± 9, PCS 47 ± 9) [71]. Sexual functioning on the SPI improved from baseline (55 ± 33) to 6 months (74 ± 32) and 12 months (80 ± 26). Body image on the Body Attitudes Questionnaire improved from baseline (61 ± 23) to 24 months (71 ± 20). These results indicate that the bulk of the improvement in QoL following hysterectomy is seen at 6 months, with further slight improvement at 2 years, although no statistical analysis was performed within each group. There were no significant differences between subtotal/total hysterectomy.

#### 3.5.3. Single Arm Cohort Studies

Three studies (with 319 patients) described single-centre single-arm cohort studies of patients undergoing hysterectomy [72,73,74]. Roberts et al. 1996 reported on 192 UK patients who underwent hysterectomy for menstrual disorders [72]. The 6–18-month follow-up with non-validated questionnaires revealed that 94% were pleased to have undergone hysterectomy, with 76% wishing they had undergone this sooner. In total, 71% reported an improvement in physical and mental health, with a majority reporting no change or improvement in sex life, and 10% reporting worsening.

Four-month outcomes of 90 patients undergoing hysterectomy for bleeding disorders including uterine fibroids were reported in a Danish study [73]. In total, 51.1% had pre-operative pelvic pain which affected daily living, with 17% women reporting this after 4 months. Only 3% of women described new onset scar pain. Those women with postoperative pain were more likely to have poorer pre-operative QoL on the SF-36 (*p* < 0.05) (numerical SF-36 data not provided).

A recent US-based study reported on sexual function and other outcomes of hysterectomy for a number of benign indications at 6 months [74]. Results for the abnormal uterine bleeding group (*n* = 37) showed no significant change in reported sexual function using the FSFI (from 24.9 ± 9.0 at baseline to 26.3 ± 7.8 at 6 months, *p* = 0.402), although there was a non-significant improvement in all domains of sexual function.

#### 3.5.4. Hysterectomy Versus Endometrial Ablation or Resection

Twelve studies reported outcomes of hysterectomy versus endometrial ablation or resection [75,76,77,78,79,80,81,82,84,85,86,87]. The UK-based Aberdeen Endometrial Ablation Trials Group randomly assigned 99 women to hysterectomy versus 105 women to endometrial resection or ablation. In total, 85 abdominal and 10 vaginal hysterectomies were performed, which was combined with BSO in 7%, and outcomes were reported at 1 year using non-validated [77] and validated questionnaires (HADS, Psychological adjustment to illness scale [88]) [78], and again at 4 years [79]. Patients who underwent hysterectomy took a mean 2–3 months until full recovery/return to work, with 7/97 (7.2%) of patients requiring over 6 months. After 1 year, 24–29% of patients had urge/stress incontinence (versus 10–40% prior), and 44% had dyspareunia (versus 47% prior). In total, 96% of patients described their health as much better/better than before surgery, and 99% were very/moderately satisfied [77]. After 6 months, the mean HADS scores showed a significant decrease in anxiety (from 9.1 ±4.0 to 4.7 ±3.7) and depression (from 5.5 [IQR 5.0–6.1] to 1.3 [IQR 1.3–1.9]), which continued at 12 months. While the effect size is statistically significant, these results are unlikely to be of “clinical importance”. Psychological adjustment to illness scale showed reduced psychological distress from a median of 9 (IQR 5.5–12) at baseline to 4 (IQR 1–7) at 6 months, which was maintained at 12 months, and improved sexual relationships from a median of 5 (IQR 2–5) at baseline to 10 (IQR 6.5–12) at 6 months, maintained at 12 months [78]. In total, 73 patients completed 4-year follow-up, which found that 93% of women were totally/generally/fairly satisfied with treatment overall, with 48% of patients having no change in sexual satisfaction, 23% reporting a slight increase and 13% reporting a decrease [79]. Premenstrual symptoms were significantly reduced in the hysterectomy compared to endometrial ablation, and there was little change in anxiety and depression scores from 1 year. Outcomes for those who underwent BSO in combination with hysterectomy are not reported separately.

Another RCT of abdominal hysterectomy versus endometrial resection was conducted in 200 women in Bristol, UK [75]. In total, 95 women underwent abdominal hysterectomy, with physical, psychological and social functioning assessed using the GHQ. At 4 months, significantly fewer women had abnormally high GHQ scores of ≥12 (from 43 to 24 women), and 94% of patients were satisfied/very satisfied; those not satisfied had high GHQ scores and several reported complications from surgery (pelvic haematoma/wound infection). Only 5% reported feeling less feminine, 94% reported improved dysmenorrhoea, and up to 46% reported improved premenstrual symptoms. Patients took a median 11 weeks off work, 4 weeks to return to daily activities and 6 weeks to resume intercourse. Generic QoL was measured at 2-year follow-up using SF-36 and VAS [76], although no baseline data are available. Hysterectomy was associated with very high scores (median 100) in five dimensions of the SF-36 (physical function, role limitation physical, role limitation emotional, social function, and pain), which was significantly greater than the endometrial resection group for pain (*p* = 0.01). VAS scores 2 years after hysterectomy were 83.8 ± 14.7, and 96% of patients were quite/very satisfied at this time.

Another UK multi-centre RCT compared abdominal/vaginal hysterectomy with endometrial ablation [80]. In total, 56 patients underwent hysterectomy (and 116 ablation), with follow-up for 3 years. After hysterectomy, patients required a mean 7.4 weeks to return to work and 5.9 weeks to resume sexual activity. All measures of psychological and social functioning (as measured by GHQ, Psychiatry outpatient mood scale, Social adjustment scale) improved after hysterectomy for the duration of follow-up (numerical data not provided), with 96% satisfaction with surgery, although there were no significant differences between treatment groups.

A cohort study from Scotland used non-validated questionnaires in women who had undergone abdominal hysterectomy and endometrial ablation [84]. Return to daily activities from hysterectomy took 8 weeks for 55% of patients, and longer than this for 17%. Hysterectomy was associated with reduced pain in 100%, reduced pre-menstrual symptoms in 88%, 84% improvement in sexual functioning (although 8% reported worsening dyspareunia), and 100% improvement in ability to work.

A 100% satisfaction at 5–24 months was also reported in a USA cohort study with 7 women undergoing hysterectomy, which was substantially higher than other patients who underwent hysteroscopic or medical treatment of HMB (43–65% satisfaction) [83].

Crosignani and colleagues reported an Italian RCT of vaginal hysterectomy versus endometrial resection [81], with 39 patients undergoing hysterectomy. Follow-up was assessed at 24 months using the SF-36, HADS and SSRS, but no baseline values are available. In total, 94.8% were satisfied/very satisfied with hysterectomy. Hysterectomy was associated with improved scores on all eight dimensions of SF-36 compared to resection, which was significant in the case of vitality (63.6 ± 20.6 for hysterectomy vs. 52.3 ± 19.3 for resection, *p* = 0.01) and social functioning (mean 80.4 ± 21.4 vs. 70.1 ± 23.0, *p* = 0.04) (although no adjustments were made for multiple comparisons). When compared with normative data for the Italian population, post-hysterectomy patients had higher scores on physical functioning, body pain, general health, vitality, social functioning, similar mental health, and slightly reduced scores for role limitations due to physical and emotional limitations. Hysterectomy was associated with significantly reduced anxiety on HADS (5.2 ± 4.0 vs. 6.8 ± 3.5 for resection, *p* = 0.03). Sexual functioning was similar between groups (48.5 ± 18.4 for hysterectomy versus 44.8 ± 14.9 for resection); only 5.7% of patients reported worsening sexual function after vaginal hysterectomy.

Tapper and colleagues reported a cohort study comparing LAVH with endometrial resection for HMB [82]. Once again, hysterectomy resulted in high rates of satisfaction: 97% were satisfied/very satisfied. Only one patient out of 40 (2.5%) complained of psychosexual difficulties at follow-up (6–31 months) (non-validated questionnaire).

The Surgical Treatments Outcomes Project for Dysfunctional Uterine Bleeding (STOP-DUB) RCT compared hysterectomy (abdominal, vaginal or laparoscopic) with endometrial ablation at 33 sites across the US and Canada [85]. EQ-5D scores show an increase from 0.678 ± 0.215 at baseline to 0.836 ± 0.168 at 6 months, which is significantly greater than the ablation group at 6 months (*p* < 0.009). These values are maintained to 24 months (0.818 ± 0.193) in the hysterectomy arm. VAS similarly improved from 61.2 ± 22.7 at baseline to 78.1 ± 21.0 at 6 months and 77.8 ± 21.1 at 24 months. SF-36 scores demonstrated that hysterectomy significantly reduced pain (61.4% of patients reported mild/moderate pain at baseline vs. 43.1% at 48 months, *p* < 0.001).

Two studies were conducted in India; the first is an RCT of vaginal hysterectomy vs. thermal balloon ablation [86], which measured outcomes including Uterine Fibroid Symptom and Quality of Life (UFS-QOL). After 6 months, all patients affected by dysmenorrhoea/pelvic pain/dyspareunia had resolution, with significant improvement (61.9% to 2.0%) in UFS-QOL, although these were not significantly different between groups (*p* > 0.49).

The second is a cohort study of hysterectomy (TAH/vaginal/TLH) and a variety of hysteroscopic surgical procedures (resection/myomectomy/polypectomy), which used SF-36 at baseline and follow-up [87]. At 6 month and 1 year post hysterectomy, there was significant improvement in all domains of SF-36 compared to baseline (*p* < 0.001), by 56.26 ± 30.57 for role limitation (emotional), 52.07 ± 9.55 for fatigue, 48.04 ± 12.91 for pain, 37.93 ± 14.45 physical health. Greater improvements were, however, seen in the hysteroscopy group compared to hysterectomy (*p* < 0.001).

### 3.6. Comparison of Hysterectomy with BSO Versus without BSO

We identified two qualitative studies (eight patients) which described RRH with BSO in all patients [32,33]. One paper describes a cohort (14 patients) of RRH with 78.6% of patients undergoing BSO [31]. Only one study (40 patients) reported that all patients underwent hysterectomy without BSO [84]. Twelve papers (of five studies) (358 patients) describe hysterectomy (as treatment of HMB/DUB) with BSO rates from 4.5 to 10.5% [63,64,65,66,67,77,78,79,80]. The remaining studies do not report BSO rates.

Papers which describe RRH with BSO noted severe and distressing menopausal symptoms for several women immediately following surgery, which impacted on their “interest, enjoyment and experience of sex” [33]. This occurred for women who did not take HRT, and for some women who did. However, for women who were older or not very sexually active, there was no negative impact. HRT was not always adequately discussed pre-operatively in this cohort [32]. In the UK study, 7/11 women were prescribed HRT and 6/7 of these had a positive experience, with “manageable” menopausal symptoms [31]. One participant would have wanted more pre-operative counselling, with explicit discussion of the potential impact on sex life. One study described a cohort who exclusively underwent hysterectomy without BSO for benign disease [84], and reported low rates (8%) of worsening sexual life after surgery. Other studies described a population cohort undergoing hysterectomy predominantly without BSO (under 4.5%) [63,64,65,66,67], and reported that hysterectomy resulted in a significant increase in hot flushes (*p* = 0.02).

### 3.7. Comparison of Route of Hysterectomy

Among studies describing RRH for EC prevention, one study (with 14 participants) describes abdominal RRH [31], with others (eight patients) describing a mixture of abdominal and laparoscopic RRH [32,33]. No comment is made on the impact of route of surgery on outcomes. Among studies of hysterectomy as treatment for HMB/DUB, one non-randomised study (with 64 patients) explicitly compared TLH vs. TAH for HMB, and found no significant differences in QoL after 6 months, except for greater improvements in social function and vitality with TLH. Five studies (202 patients) report exclusively on TAH ([70,71,75,76,84], one study (39 patients) exclusively on LAVH [81], one study (40 patients) exclusively on TLH [82], one study (20 patients) exclusively on vaginal hysterectomy [86], with the remainder reporting a mixture of routes or not specifying. Comparisons between routes of hysterectomy are limited, due to the heterogeneous nature of outcomes reported.

## 4. Discussion

### 4.1. Findings

This systematic review identified extremely limited evidence on the QoL of women after RRH. Available research is in the form of two pilot qualitative studies, a very small mixed-methods study using validated questionnaires, and a slightly larger but small size quantitative study which used non-validated questionnaires. All of these were conducted in women with Lynch Syndrome. Together, these conclude that that RRH seems to reduce cancer worry, which may be greater for those with superior post-operative QoL, and there appears to be high levels of satisfaction with no decision-regret. As expected, pre-menopausal RRH with BSO causes significant menopausal symptoms if HRT is not prescribed. Some women reported insufficient counselling around HRT; however, those who did take HRT reported far fewer menopausal symptoms and higher satisfaction. These two cohort studies had moderate risk of bias, particularly due to the small sample size and lack of representativeness of the wider population of women who may be at increased EC risk. Some women at increased EC risk may also be at increased risk of breast cancer (BC) and/or may have had BC themselves, for example women with PTEN syndrome [89]. PTEN women with BC may not be able to take HRT following RRH for EC.

We considered a surrogate population model of hysterectomy as a treatment for selected benign gynaecological conditions, in order to provide additional evidence which may be used to estimate the likely impact of RRH on QoL. These studies show very high satisfaction rates with hysterectomy. For women with HMB/DUB, hysterectomy improved their overall QoL. Most women reported an improvement or no change in their sexual function/satisfaction, and in urinary urge/stress incontinence, with only very small numbers reporting a worsening in sexual functioning or stress incontinence following surgery. Hysterectomy reduced the incidence of premenstrual symptoms, pelvic pain, and in some studies scores of anxiety/depression. Following abdominal or vaginal hysterectomy, patients required a mean of 6 weeks to resume intercourse following surgery, and 2–3 months to return to full work. The bulk of the improvements in QoL are seen at 6 months post hysterectomy, although small improvements continue up to 2–5 years. These RCTs and cohort studies had in some cases moderate risk of bias, due to lack of clarity regarding blinding, and assessments of outcomes.

### 4.2. Strengths and Weaknesses 

This is the first and to our knowledge the only systematic review investigating QoL outcomes after RRH in women at increased EC risk. Our methodology followed a prospectively registered protocol, and was conducted using the highest standards according to PRISMA guidelines. We provide a clear summary of outcomes and validated questionnaires that have been used in included studies, which may assist other researchers designing future studies.

We recognise some limitations with our review. Due to the lack of data available on RRH, there can be only limited confidence in any results obtained. Furthermore, the population undergoing RRH were women with Lynch Syndrome who are generally at high risk of EC as well as ovarian and colorectal cancer. Thus results, particularly regarding reduction in cancer worry and satisfaction may not be generalisable to women who are only at increased risk of EC, including those at moderate EC risk, and women without additional risk of secondary cancers at other sites.

To address the lack of evidence on RRH, an alternative (surrogate) population model was also used, where the type of hysterectomy may have a similar theoretical impact on QoL. This study is not intended to be a systematic review of QoL after any hysterectomy, or all benign hysterectomies, but rather to provide additional evidence which may help in estimating the QoL after RRH. Although this alternative population was deliberately restricted in scope, variation was nevertheless seen in the incidence of pre-operative benign pathology and symptomatology, and the surgical procedure performed. Given that QoL is a very heterogeneous topic there were a variety of outcomes measured and several tools were used to obtain these, such that no meaningful quantitative synthesis could be performed. There are challenges and limitations in interpreting and applying this evidence from such treatment hysterectomies for patients undergoing risk reduction, as discussed below.

### 4.3. Interpretation

Women with HMB/DUB were found to have lower generic QoL than the age-matched general population, which is seen in other studies [90,91,92]. In all reporting studies, the QoL of patients improved following treatment by 6 months, and remained elevated for 5–10 years, in some studies higher than the age-matched population reference. This was particularly so for women who underwent hysterectomy without BSO. This reflects improved QOL in symptomatic women following an effective treatment. This is less likely to be the case with RRH, where an otherwise asymptomatic patient (with increased cancer risk) undergoes a major surgical procedure. A critical issue for women considering RRH is not whether their QoL will improve, but whether there will be an impairment in QoL following surgery, and if so, to what degree. To address this, we specifically sought evidence of the impact on selected symptoms that may be affected by hysterectomy, such as pelvic floor and sexual function.

A distinction should be drawn between the impact of RRH (for EC prevention) and that of BSO (for OC prevention). Whilst women with Lynch Syndrome make up the vast majority of patients undergoing RRH, and most are recommended to have RRH-BSO due to their heightened OC risk [14], not all EC prevention must be combined with OC prevention. Women with *PMS2* pathogenic variants do not require BSO. While cost-effectiveness analysis of alternative strategies such as two-step surgery has been undertaken in Lynch Syndrome, there are no prospective trials evaluating this strategy and thus no available QOL outcomes [13]. Furthermore, women with *PTEN* pathogenic variants, a family history of EC, type 2 diabetes and/or obesity may benefit from RRH without BSO for increased EC (without OC) risk. Models are currently being developed for predicting personalised EC risk [10,11,93,94], following similar successful approaches in breast cancer/OC prediction [95,96]. With the rising incidence of EC and associated EC risk-factors, and ability to provide a personalised EC-risk estimate, many more people may be eligible for EC (but not OC) risk-reduction in future.

BSO causes a substantial component of the reported adverse consequences following pre-menopausal RRH for Lynch Syndrome, in particular a negative impact on menopausal symptoms and sexual function. These findings are in keeping with our recent systematic review of QoL following BSO for OC prevention. HRT can partially mitigate these effects ameliorating symptoms and improving sexual function. Additionally, it can prevent long-term consequences of a premature menopause such as cardiovascular risk/mortality and osteoporosis [24]. HRT until the age of natural menopause is not associated with an increased breast cancer risk, even in carriers of breast cancer-associated susceptibility genes, [24,97] and is recommended in this situation [25]. Lynch Syndrome, currently the commonest reason for RRH, is not associated with an increased risk of breast cancer [98]. Such issues should be part of a detailed counselling process for women at increased risk of OC as well as EC. We would advocate that risk-reduction services offer routine access to menopause specialists as part of pre-operative counselling and post-operative follow-up, as it is known that HRT compliance and service satisfaction is higher in a specialist multi-disciplinary setting [24,99].

In considering RRH (without BSO) for EC prevention alone, our review found one study which reported a negative impact on ovarian function, as assessed by serum follicular stimulating hormone and the KI [65]. Other studies support this, and have found decreased anti-müllerian hormone levels following hysterectomy which is not explained by a lower pre-operative ovarian reserve [100,101]. This is consistent with a reported 2–6 year earlier age of menopause in women undergoing hysterectomy alone [100,102,103,104]. This may be due to disrupted paracrine signalling, or a reduction in blood flow after ligation of the uterine arteries. Such issues should be mentioned during RRH counselling.

Our review found that a majority of women in included studies reported an improvement or no change in urinary incontinence symptoms, with a very small number reporting any worsening. Other studies find no impact of hysterectomy (as treatment of benign disease) on pelvic function at 1 year [105], or 10 years [106], findings supported by a systematic review of urodynamic outcomes and urinary symptoms [107]. In contrast, other large studies with extensive follow-up, including Swedish registry-based cohort studies, have shown an increased risk of urinary tract infections, stress urinary incontinence, and subsequent surgery for stress urinary incontinence after hysterectomy [108,109,110]. This issue is unresolved; differences may be explained by the prevalence of pelvic floor dysfunction in cohorts undergoing hysterectomy for benign conditions, due to the nature of that gynaecological condition as well as co-morbidities, body mass index and age, which highlights the need for research into the specific population undergoing EC risk-reduction. For women considering RRH, it may be appropriate for counselling to take these individualised factors into account regarding the likely impact on pelvic floor function, in the absence of robust evidence.

Whilst a negative impact on sexual function was found, particularly from studies of women undergoing RRH with BSO and without post-operative HRT, improvements in sexual function were seen in studies of hysterectomy as treatment of HMB/DUB. Similarly, concomitant hysterectomy was not associated with sexual dysfunction in studies of *BRCA* pathogenic variant carriers undergoing risk-reducing BSO [111,112]. In our review, hysterectomy (without BSO) was not associated with high rates of de novo impaired sexual function or satisfaction, a finding supported by a recent systematic review and meta-analysis [113]. For women having RRH with BSO, adequate pre-operative counselling about any potential impact on sexual function and HRT options is associated with improved satisfaction and reduced sexual problems [114].

This study was not able to draw meaningful conclusions on the impact of route of RRH on QoL, and found little to no evidence of any difference between abdominal or minimally invasive approaches for RRH. This reflects the lack of meaningful data on this issue. However, inferences may be drawn from the non-risk reducing hysterectomy literature. A retrospective registry study [115] found that route of hysterectomy had only a small impact on the risk of subsequently undergoing pelvic organ prolapse surgery. In our review, patients who underwent (predominantly abdominal) hysterectomy as treatment of HMB/DUB required a mean of 2–3 months to return to work; other studies reported that vaginal/TLH/TRH significantly reduces this convalescence [116,117], by a mean 8 days. Systematic reviews have shown that TLH is associated with equal or better quality of life than TAH in the first 6 weeks [52,118], but long-term comparative data are lacking.

Post-operative complications may occur in under 10% of patients following hysterectomy [119,120], although a major predictor is the presence of severe endometriosis, which we excluded as this is not representative of typical RRH. Whilst our review did not focus on post-operative complications, these can be directly reflected in summary QoL outcomes, and therefore reducing complication rates may be expected to improve QoL in patients.

Together, these issues (of menopausal impact, sexual and pelvic floor function) further highlight why research into QoL following RRH is required, separate to that of hysterectomy as treatment of gynaecological disease. Further research should use validated QoL questionnaires including EQ-5D, which will also enable calculation of utility scores. A gold-standard study would be prospectively designed with baseline and post-operative questionnaires; however, due to low numbers of women undergoing RRH this would need to be multi-centre with significant funding and timelines to recruit sufficiently, and so cross-sectional or retrospective studies may be more cost-efficient. Given the challenge of separating the impact of RRH from RRH-BSO, novel or alternative study designs may be justified in determining the utility scores of each [28,121].

## 5. Conclusions

This systematic review has found limited evidence of QoL after RRH. RRH is associated with high satisfaction and reduction in cancer worry, and low decision regret. Data were restricted to women with Lynch Syndrome and lacking for women with moderate EC-risk or increased levels of EC-risk alone which are below the extremely high-risk levels seen in women with Lynch Syndrome. Patients describe the major impact following RRH-BSO arising from a premature menopause, leading to menopausal symptoms and impacted sexual function, which can be severe without and tolerable with HRT. By using an alternative population model of hysterectomy as treatment of HMB/DUB, hysterectomy (usually without BSO) was found to improve QoL, and result in high rates of improvement/no change in sexual function or urinary symptoms, and very low rates of a de novo impact. The applicability of these results to patients considering EC risk-reduction is uncertain, and hence further research using validated questionnaires is required to better determine QoL following RRH at varying levels of EC risk, and allow for greater confidence in health-economic modelling of EC prevention.

## Figures and Tables

**Table 1:** Characteristics of included studies.

First Author, Year	Country	Study Design	Hysterectomy Sample Size at Follow-up (*n*)	Age of Hysterectomy Participants (Years) (mean ± SD Unless Specified)	Follow-up (Months)	Population/Indication	BSO	Route of Hysterectomy
Risk-reducing hysterectomy								
Moldovan 2015 [31]	UK	Qualitative and cohort	14 (quantitative) 11 (qualitative)	52.5	12–144	Lynch Syndrome + family history	11/14 (78.6%)	TAH
Etchegary 2015 [32]	Canada	Qualitative	8	49 (range 33–64)	1.5–96 (mean 42)	Lynch Syndrome	yes	LAVH (6), TAH (2)
Etchegary 2018 [33]	Canada	Qualitative	8	49 (range 33–64)	36–120	Lynch Syndrome	yes	TAH and TLH
Kalamo 2020 [34]	Finland	Cohort	42	56.9 (range 43–72)	12–456	Lynch Syndrome	not reported	not reported
Treatment of heavy menstrual bleeding								
*Hysterectomy* vs. *hormonal treatment*								
Hurskainen 2001 [63]	Finland	RCT: hysterectomy vs. LNG-IUS	112	43.1 ± 3.5	12	HMB	5/112 (4.5%)	TAH 21, vaginal 30, TLH 56
Hurskainen 2004 [64]	Finland	RCT: 5-year follow-up of Hurskainen 2001	115	43.1 ± 3.5	60	HMB	5/112 (4.5%)	TAH 21, vaginal 30, TLH 56
Halmesmäki 2004 [65]	Finland	RCT: Menopausal impact of Hurskainen 2001	107	43.1 ± 3.5	12	HMB	5/112 (4.5%)	TAH 21, vaginal 30, TLH 56
Halmesmäki 2007 [66]	Finland	RCT: 5-year follow-up of Hurskainen 2001	117	43.1 ± 3.5	60	HMB	5/112 (4.5%)	TAH 21, vaginal 30, TLH 56
Heliövaara-Peippo 2013 [67]	Finland	RCT: 10-year follow-up of Hurskainen 2001	117	43.1 ± 3.5	120	HMB	5/112 (4.5%)	TAH 21, vaginal 30, TLH 56
Kuppermann 2004 [68]	USA	RCT: hysterectomy vs. oral medical treatment	28	42.0 ± 4.6	24	DUB	“discouraged” but not reported	TAH or vaginal
Adigüzel 2017 [69]	Turkey	Cohort: TLH vs. TAH vs. LNG-IUS	64	48.4 ± 4.4	6	HMB	not reported	29 TAH, 35 TLH
*Comparison of total* vs. *subtotal*								
Learman 2003 [70]	USA	RCT: total vs. subtotal	64	41.8 ± 5.2	24	DUB	not reported	TAH
Kuppermann 2005 [71]	USA	RCT: 2-year outcomes of Learman 2003	67	41.8 ± 5.2	24	DUB	not reported	TAH
*Cohort/other*								
Roberts 1996 [72]	UK	Cohort	192	43.0 (range 21–56)	6–18	DUB	not reported	242 TAH, 5 vaginal, 17 LAVH
Brandsborg 2009 [73]	Denmark	Cohort	90	46 (median) (range 32–71)	4	DUB/fibroids	not reported	TAH, vaginal, LAVH (numbers not reported)
Till 2022 [74]	USA	Cohort: different indications of hysterectomy	37	44.2 ± 6.9	6	DUB/HMB	not reported	TLH (70%), TAH (11%), vaginal (19%)
*Hysterectomy* vs. *endometrial ablation/resection*								
Dwyer 1993 [75]	UK	RCT: hysterectomy vs. TCRE	95	40.6	4	HMB	up to 14/95 (10.5%)	TAH
Sculpher 1996 [76]	UK	RCT: 2-year follow-up of Dwyer 1993	73	40.6	34 (mean)	HMB	up to 14/95 (10.5%)	TAH
Pinion 1994 [77]	UK	RCT: hysterectomy vs. TCRE vs. ablation	95	40.3 ± 5.2	12	DUB	6/85 (7.1%)	85 TAH 10 vaginal
Alexander 1996 [78]	UK	RCT: 1-year follow-up of Pinion 1994	85	40.3 ± 5.2	12	DUB	6/85 (7.1%)	85 TAH 10 vaginal
Aberdeen Trials Group 1999 [79]	UK	RCT: 4-year follow-up of Pinion 1994	72	40.3 ± 5.2	48	DUB	in 6/85 (7.1%)	85 TAH 10 vaginal
O’Connor 1997 [80]	UK	RCT: hysterectomy vs. TCRE	56	39.4 ± 4.8	12–36	HMB	0.04	TAH/vaginal
Crosignani 1997 [81]	Italy	RCT: hysterectomy vs. TCRE	39	45 (median) (IQR 42–48.5)	24	HMB	not reported	LAVH
Tapper 1998 [82]	Finland	Cohort: hysterectomy vs. TCRE	40	43 ± 4.0	6–31	HMB	not reported	TLH
Tjarks 2000 [83]	USA	Cohort: hysterectomy vs. polypectomy ± ablation vs. medical treatment	7	49 (range 28–74)	5–24	DUB/HMB	not reported	not reported
Mousa 2001 [84]	UK	Cohort: hysterectomy vs. ablation	40	41 (median) (range 30–48)	32 (mean) (range 18–55)	HMB	no	TAH
Dickersin 2007 [85]	USA, Canada	RCT: hysterectomy vs. ablation	107	not reported	24	DUB	18/114 (7.0%)	(TAH/vaginal/TLH-numbers not reported)
Jain 2016 [86]	India	RCT: hysterectomy vs. thermal balloon ablation	20	44 ± 2.0	24	DUB	not reported	vaginal
Selvanathan 2019 [87]	India	Cohort: hysterectomy vs. hysteroscopic surgery	176	40.2 ± 4.3	48	DUB/HMB	not reported	116 TAH, 27 vaginal, 32 TLH

BSO: Bilateral salpingo-oophorectomy, DUB: Dysfunctional uterine bleeding, HMB: Heavy menstrual bleeding, HRT: Hormone replacement therapy, IQR: Interquartile range, LAVH:Laparoscopic assisted vaginal hysterectomy, LNG-IUS: levonorgestrel releasing intra-uterine system, QoL: Quality of life, RCT: Randomised control trial, SD: Standard deviation, TAH: Total abdominal hysterectomy, TCRE: Transcervical resection of endometrium, TLH: Total laparoscopic hysterectomy.

**Table 2:** Summary of main findings.

First Author, Year	Relevant Findings for Hysterectomy Group
Risk-reducing hysterectomy	
Moldovan 2015 [31]	No decision regret, reduced cancer worry. Few patients had menopausal symptoms impacting QoL, associated with poorer general health and psychological distress.
Etchegary 2015 [32]	Surgery alleviated cancer fear, no decision regret. Menopausal symptoms for women not on HRT
Etchegary 2018 [33]	High satisfaction in women who took HRT. Severe menopausal symptoms in those who did not take HRT.
Kalamo 2020 [34]	Generally satisfied with RRH. 45% still had a strong fear of cancer.
Treatment of heavy menstrual bleeding	
*Hysterectomy* vs. *hormonal treatment*	
Hurskainen 2001 [63]	Improved generic QoL, general health, reduced anxiety and depression, improved sexual satisfaction
Hurskainen 2004 [64]	Greater satisfaction, reduced anxiety/depression, better general health. Worse sexual satisfaction
Halmesmäki 2004 [65]	No signicant changes in overall menopausal index scores, although increase in vasomotor symptoms
Halmesmäki 2007 [66]	Increased self and partner sexual satisfaction, reduced sexual problems compared to baseline and to the Mirena
Heliövaara-Peippo 2013 [67]	Increase in overall QoL and sexual satisfaction until 5 years, decrease in QoL including sexual satisfaction between 5 to 10 years back to baseline
Kuppermann 2004 [68]	Improved QoL, satisfaction, symptom resolution, sexual desire and health distress. Improvements seen at 6 months, maintained at 2 years
Adigüzel 2017 [69]	Hysterectomy generally better health, social function better with TLH, no change in mental health parameters
*Comparison of hysterectomy type*	
Learman 2003 [70]	Improvement in pain and urinary symptoms
Kuppermann 2005 [71]	Improved sexual functioning, general QoL, and body image, no differences between groups
*Cohort/other*	
Roberts 1996 [72]	improved physical and mental health, majority had no change in sex life
Brandsborg 2009 [73]	Decrease in pain at 4 months. Those with low pre-operative QoL more likely to have ongoing post-operative pain
Till 2022 [74]	No significant change in sexual function (although a trend towards improved function)
*Hysterectomy* vs. *endometrial ablation/resection*	
Dwyer 1993 [75]	Higher satisfaction, fewer pre-menstrual symptoms
Sculpher 1996 [76]	Very high satisfaction. Evidence of improved QoL compared to resection
Pinion 1994 [77]	High satisfaction and improvement in symptoms
Alexander 1996 [78]	Reduced anxiety, depression and improved sexual relationships
Aberdeen Trials Group 1999 [79]	Generally better health, fewer pre-menstrual symptoms, high satisfaction
O’Connor 1997 [80]	Very high satisfaction, improvement in general quality of life and psychosocial functioning
Crosignani 1997 [81]	Greater satisfaction, greater social functioning and vitality, lower anxiety, similar sexual functioning
Tapper 1998 [82]	High satisfaction
Tjarks 2000 [83]	Hysterectomy had 100% satisfaction, greater than other treatment modalities
Mousa 2001 [84]	High satisfaction, improvement in general health inc sexual function, PMS
Dickersin 2007 [85]	Improved generic QoL, particularly reduced pain and fatigue compared to ablation
Jain 2016 [86]	Resolution of dysmenorrhea and pelvic pain, increase in generic QoL
Selvanathan 2019 [87]	Improvement in all QoL domains, greater in hysteroscopic group vs. hysterectomy

HRT: Hormone replacement therapy, PMS: Premenstrual symptoms, QoL: Quality of life, RRH: Risk reducing hysterectomy, TLH: Total laparoscopic hysterectomy.

## Data Availability

All data relating to this study are contained within the manuscript and Supplementary Materials.

## Supplementary Materials

The following supporting information can be downloaded at: https://www.mdpi.com/article/10.3390/cancers14235832/s1, File S1: Search strategy; Table S1: Outcomes Measured; Table S2: NICE quality appraisal checklist for qualitative studies; Table S3: Jadad scale for randomised controlled trials; Table S4: Newcastle Ottawa Scale for cohort studies.
