# Supplementary material for: Quality of Life after Risk-Reducing Hysterectomy for Endometrial Cancer Prevention: A Systematic Review
Source: Cancers (Basel). 2022 Nov 26;14(23):5832. doi: 10.3390/cancers14235832 (PMC9736914; doi:10.3390/cancers14235832)
Supplement: Supplementary file 1 [file cancers-14-05832-s001.zip › cancers-2009507-supplementary.pdf]

File S1

Search Strategy

Quality of life after risk-reducing hysterectomy: a systematic review

Date of searches: 19 July 2022

PubMed and Medline:

("colorectal neoplasms, hereditary nonpolyposis"[MeSH Terms] OR ("colorectal"[All Fields] AND "neoplasms"[All Fields] AND "hereditary"[All Fields] AND "nonpolyposis"[All Fields]) OR "hereditary nonpolyposis colorectal neoplasms"[All Fields] OR ("lynch"[All Fields] AND "syndrome"[All Fields]) OR "lynch syndrome"[All Fields] OR ("endometrial hyperplasia"[MeSH Terms] OR ("endometrial"[All Fields] AND "hyperplasia"[All Fields]) OR "endometrial hyperplasia"[All Fields]) OR ("menorrhagia"[MeSH Terms] OR "menorrhagia"[All Fields] OR ("heavy"[All Fields] AND "menstrual"[All Fields] AND "bleeding"[All Fields]) OR "heavy menstrual bleeding"[All Fields]) OR "risk reducing surgery"[All Fields] OR "preventive surgery"[All Fields])

AND ("hysterectomy"[MeSH Terms] OR "hysterectomy"[All Fields] OR "hysterectomies"[All Fields])

AND ("quality of life"[MeSH Terms] OR ("quality"[All Fields] AND "life"[All Fields]) OR "quality of life"[All Fields] OR "bladder function"[All Fields] OR "sexual function"[All Fields] OR "decision regret"[All Fields] OR ("menopause"[MeSH Terms] OR "menopause"[All Fields] OR "menopausal"[All Fields] OR "menopaused"[All Fields] OR "menopauses"[All Fields]) OR "cancer worry"[All Fields] OR ("personal satisfaction"[MeSH Terms] OR ("personal"[All Fields] AND "satisfaction"[All Fields]) OR "personal satisfaction"[All Fields] OR "satisfaction"[All Fields] OR "satisfactions"[All Fields] OR "satisfaction s"[All Fields]) OR ("psychosocial"[All Fields] OR "psychosocially"[All Fields]))

Translations

Lynch syndrome: "colorectal neoplasms, hereditary nonpolyposis"[MeSH Terms] OR ("colorectal"[All Fields] AND "neoplasms"[All Fields] AND "hereditary"[All Fields] AND "nonpolyposis"[All Fields]) OR "hereditary nonpolyposis colorectal neoplasms"[All Fields] OR ("lynch"[All Fields] AND "syndrome"[All Fields]) OR "lynch syndrome"[All Fields]

endometrial hyperplasia: "endometrial hyperplasia"[MeSH Terms] OR ("endometrial"[All Fields] AND "hyperplasia"[All Fields]) OR "endometrial hyperplasia"[All Fields]

heavy menstrual bleeding: "menorrhagia"[MeSH Terms] OR "menorrhagia"[All Fields] OR ("heavy"[All Fields] AND "menstrual"[All Fields] AND "bleeding"[All Fields]) OR "heavy menstrual bleeding"[All Fields]

hysterectomy: "hysterectomy"[MeSH Terms] OR "hysterectomy"[All Fields] OR "hysterectomies"[All Fields]

quality of life: "quality of life"[MeSH Terms] OR ("quality"[All Fields] AND "life"[All Fields]) OR "quality of life"[All Fields]

menopause: "menopause"[MeSH Terms] OR "menopause"[All Fields] OR "menopausal"[All Fields] OR "menopaused"[All Fields] OR "menopauses"[All Fields]

satisfaction: "personal satisfaction"[MeSH Terms] OR ("personal"[All Fields] AND "satisfaction"[All Fields]) OR "personal satisfaction"[All Fields] OR "satisfaction"[All Fields] OR "satisfactions"[All Fields] OR "satisfaction's"[All Fields]

psychosocial: "psychosocial"[All Fields] OR "psychosocially"[All Fields]

Embase:

('hereditary nonpolyposis colorectal cancer' OR 'lynch syndrome' OR 'lynch syndrome ii' OR 'endometrium hyperplasia' OR 'menorrhagia' OR 'risk reducing surgery' OR 'risk reduction' OR 'preventive medicine')

AND

('hysterectomy'/exp OR hysterectomy)

AND

('quality of life' OR 'bladder function' OR 'sexual function' OR 'decision regret' OR 'cancer worry scale' OR 'satisfaction')

Table S1. Outcomes measured.

Study		Outcomes measured																
First author	Year	Qualitative	Generic QoL			Menopausal		Sexual function				Psychological					Satisfaction	Non-validated
			SF-36	EQ-5D	VAS	KI	MENQOL	MSS	SSRS	SPI	FSFI	GHQ	BDI	HADS	STAI	Other		
**Risk-reducing hysterectomy**																		
Etchegary	2015																	
Etchegary	2018																	
Moldovan	2015															CWS, IES		
Kalamo	2020																	
**Treatment of heavy menstrual bleeding**																		
Hysterectomy vs Mirena coil/ medication																		
Hurskainen	2001																	
Hurskainen	2004																	
Halmesmäki	2004																	
Halmesmäki	2007																	
Heliövaara-Peippo	2013																	
Kupper-mann	2004															BAQ, SPI		
Adigüzel	2017																	
Comparison of total vs subtotal hysterectomy																		
Learman	2003																	
Kupper-mann	2005															BAQ, SPI		
Cohort																		
Roberts	1996																	
Brandsborg	2009																	
Till	2022															PROMIS		
Hysterectomy vs endometrial ablation/resection																		
Pinion	1994																	
Alexander	1996															PAIS		
Aberdeen Trials Group	1999															PAIS		
Dwyer	1993																	
Sculpher	1996																	
Mousa	2001																	
O'Connor	1997															POMS, SAS		

Tjarks	1998																	
Crosignani	1997																	
Tapper	1998																	
Dickersin	2007																	
Jain	2016																	
Selvanathan	2019																	

Table S2: NICE quality appraisal checklist for qualitative studies

	**Theoretical**		**Design**	**Data collection**	**Trustworthiness**			**Analysis**						**Ethics**	**Overall**
**Study**	**Qualitative appropriate?**	**Study clear?**	**Defensible methodology?**	**Collected appropriately?**	**Researcher clearly described?**	**Context described?**	**Methods reliable?**	**Rigorous?**	**Data rich?**	**Reliable?**	**Convincing?**	**Findings relevant?**	**Conclusions adequate?**	**Clearly reported?**	**How well conducted?**
Etchegary 2015	Yes	Yes	Yes	Yes	No	Yes	Not sure	Yes	Yes	Yes	Yes	Yes	Yes	Yes	++
Etchegary 2018	Yes	Yes	Yes	Yes	No	Yes	Yes	Yes	Yes	Yes	Yes	Yes	Yes	Yes	++
Moldovan 2015	Yes	Yes	Yes	Yes	No	No	Not sure	Yes	Yes	Yes	Yes	Yes	Yes	Yes	++

Table S3 Jadad scale for randomised controlled trials

Author	Randomisation	Blinding	Account of all patients	Total
Hurskainen 2001	🟢	🔴	🟢	**3**
Kuppermann 2004	🟡	🔴	🟢	**2**
Learman 2003	🟢	🟡	🟢	**4**
Pinion 1994	🟡	🔴	🟢	**2**
Dwyer 1993	🟢	🔴	🟢	**3**
O'Connor 1997	🟢	🔴	🟢	**3**
Crosignani 1997	🟢	🟡	🟢	**4**
Dickersin 2007	🟢	🔴	🟢	**3**
Jain 2016	🟢	🟡	🟢	**4**

Table S4: Newcastle Ottawa Scale for cohort studies

**Study**	**Representative-ness of cohort**	**Selection of non-exposed cohort**	**Ascertainment of exposure**	**Outcome not at start**	**Comparability**	**Assessment of outcome**	**Sufficient follow up**	**Adequacy of follow up**	**Total**
**Risk-reducing hysterectomy**									
Moldovan 2015	1	0	1	0	0	0	1	1	4
Kalamo 2020	1	1	1	0	0	0	1	1	5
**Treatment of HMB/DUB**									
Adigüzel 2017	1	1	1	1	0	0	1	1	6
Roberts 1996	1	0	1	0	0	0	1	1	4
Brandsborg 2009	1	0	1	1	0	0	0	1	4
Till 2022	1	0	1	1	0	0	1	0	4
Mousa 2001	1	1	1	0	0	0	1	1	5
Tjarks 2000	1	1	1	1	0	0	1	1	6
Tapper 1998	1	1	1	0	2	0	1	1	7
Selvanathan 2019	1	1	1	1	0	0	1	0	5
